# Assessment of the epi-pericardial fibrotic substrate by collagen-targeted probes

**DOI:** 10.1038/s41598-022-08688-x

**Published:** 2022-04-05

**Authors:** Martin Ezeani, Asif Noor, Paul S. Donnelly, Be’eri Niego, Christoph E. Hagemeyer

**Affiliations:** 1 NanoBiotechnology Laboratory, Australian Centre for Blood Diseases, Central Clinical School, Faculty of Medicine, Nursing and Health Sciences, Monash University, Melbourne, VIC 3004 Australia; 2 School of Chemistry, Bio21 Molecular Science and Biotechnology Institute, University of Melbourne, Melbourne, VIC 3010 Australia

**Keywords:** Cardiac hypertrophy, Peptide delivery

## Abstract

The identification of the fibrotic arrhythmogenic substrate as a means of improving the diagnosis and prediction of atrial fibrillation has been a focus of research for many years. The relationship between the degree of atrial fibrosis as a major component of atrial cardiomyopathy and the recurrence of arrhythmia after AF ablation can correlate. While the focus in identification and characterisation of this substrate has been centred on the atrial wall and the evaluation of atrial scar and extracellular matrix (ECM) expansion by late gadolinium-enhancement (LGE) on cardiac magnetic resonance imaging (CMRI), LGE cannot visualise diffuse fibrosis and diffuse extravasation of gadolinium. The atrial pericardium is a fine avascular fibrous membranous sac that encloses the atrial wall, which can undergo remodelling leading to atrial disease and AF. Nevertheless, little attention has been given to the detection of its fibrocalcification, impact on arrhythmogenesis and, most importantly, on the potential prothrombotic role of epi-pericardial remodelling in generation of emboli. We have recently reported that tracers against collagen I and IV can provide a direct assessment of the ECM, and thus can estimate fibrotic burden with high sensitivity. Here, we show the ability of these optical tracers to identify epi-pericardial fibrosis, as well as to demonstrate subtle interstitial fibrosis of the atrial wall in a mouse model of beta-2-adrenergic receptor (β_2_-AR) cardiac overexpression.

## Introduction

Atrial cardiomyopathy is an independent risk factor of AF-associated stroke and a determinant of arrhythmia progression. It is defined as ‘any complex of structural, architectural, contractile or electrophysiological changes affecting the atria that might have the potential to produce clinically-relevant manifestations’^1^, and was first used as a substrate for AF in 1997^2^. As a pathology-producing atrial muscle disease, a major component of which is structural remodelling, the impact of atrial myoendocardial-centric fibrosis on the occurrence of AF has been a major focus^3^. In contrast to the atrial wall pathobiology, not much attention has been given to fibrosis of the atrial epi-pericardium, which can infiltrate the myo-endocardium of the atria and favour atrial wall functional dissociation of electrical activity, contributing to wavebreak, rotor formation, thrombus generation and stroke development. To improve the diagnosis and prediction of atrial fibrillation, the identification and characterisation of the epi-pericardial fibrotic calcification as a component of the fibrotic arrhythmogenic substrate is crucial. This includes the acquisition of fibrous and ECM information on both the macroscopic and molecular scales to increase the diagnostic ability and guide treatment options in modern personalised medicine.

Considerable attention has been given to the use of non-invasive imaging technology to detect cardiac fibrosis to improve AF risk prediction, so that high-risk patients can be better identified, enabling intervention before advanced clinical state. Available data on single centre and multi-centre studies of LGE fibrosis imaging are witnessing a remarkable increase since 2009^4^. The DECAAF study and others report that LGE cardiac magnetic resonance imaging can visualise atrial fibrosis and perhaps represents a good predictor for the outcome of AF interventional management^5–7^.

Undoubtedly, LGE is making inroads (becoming a gold standard modality) in facilitating the diagnosis of atrial fibrosis; however, LGE is not efficient at visualising diffuse fibrosis, especially when viable myocardium and reactive fibrosis are involved. Most importantly, the pericardium is thin, and the atrial wall as well as the left atria is located posterior to the heart, complicating its imaging further. Identification of fibrosis for better disease management requires a technique that can track subclinical fibrotic arrhythmogenic substrate. Therefore, direct molecular imaging would be more adequate to access the atrial fibro-pericardial lesions and its influence on accompanying AF.

Fluorescence illumination imaging technologies have grown rapidly both in physiological and medical sciences since 1924, when the endogenous porphyrins autofluorescence was reported in tumours lit with ultraviolet light^8^. The first use of fluorescein by green light emission to enhance the visualisation of brain tumours was observed in 1948^9^ and red fluorescence of tumours was shown following intravenous injection of porphyrins in 1942^10^. Fluorescence imaging modality has spiked the innovation of technology and genetic reporter systems, as well as ultra-modern imaging systems, providing information on protein and gene expression and molecular interaction for both clinical and experimental observations^11^. In this context, the use of a fluorescent probe that can directly target and bind to collagens, resulting in a high target-to-background signal that is dependent on pathogenesis and disease state, may be able to assist in diagnosis.

Our newly developed molecular tracers^12^ target and bind to collagens. These tracers were deployed to quantify myocardial interstitial diffuse fibrosis to demonstrate near-infrared (NIR) visualization of atrial fibro-pericardial lesions at the level of active or less organised lesional collagen deposition. This approach was evaluated in a mouse model overexpressing beta-2-adrenergic receptor (β_2_-AR) with dominant epi-pericardial fibrosis of the atria. In this study, we showed atrial epi-pericardial fibrosis relative to the endocardial fibrosis, and then characterised the abundance of respective collagen types within the atrial layers. The report demonstrates how sulfo-Cy5.5-labelled peptides, using NIR illumination, are capable of visualising atrial pericardial fibrosis in β_2_-AR mice, supporting the application of these new probes in non-invasive imaging.

## Results

### Atrial wall to epi-pericardial collagen expression in β_2_-AR mice: evidence for atrial, pericardial-dominant fibrotic remodelling

**Figure 1:**
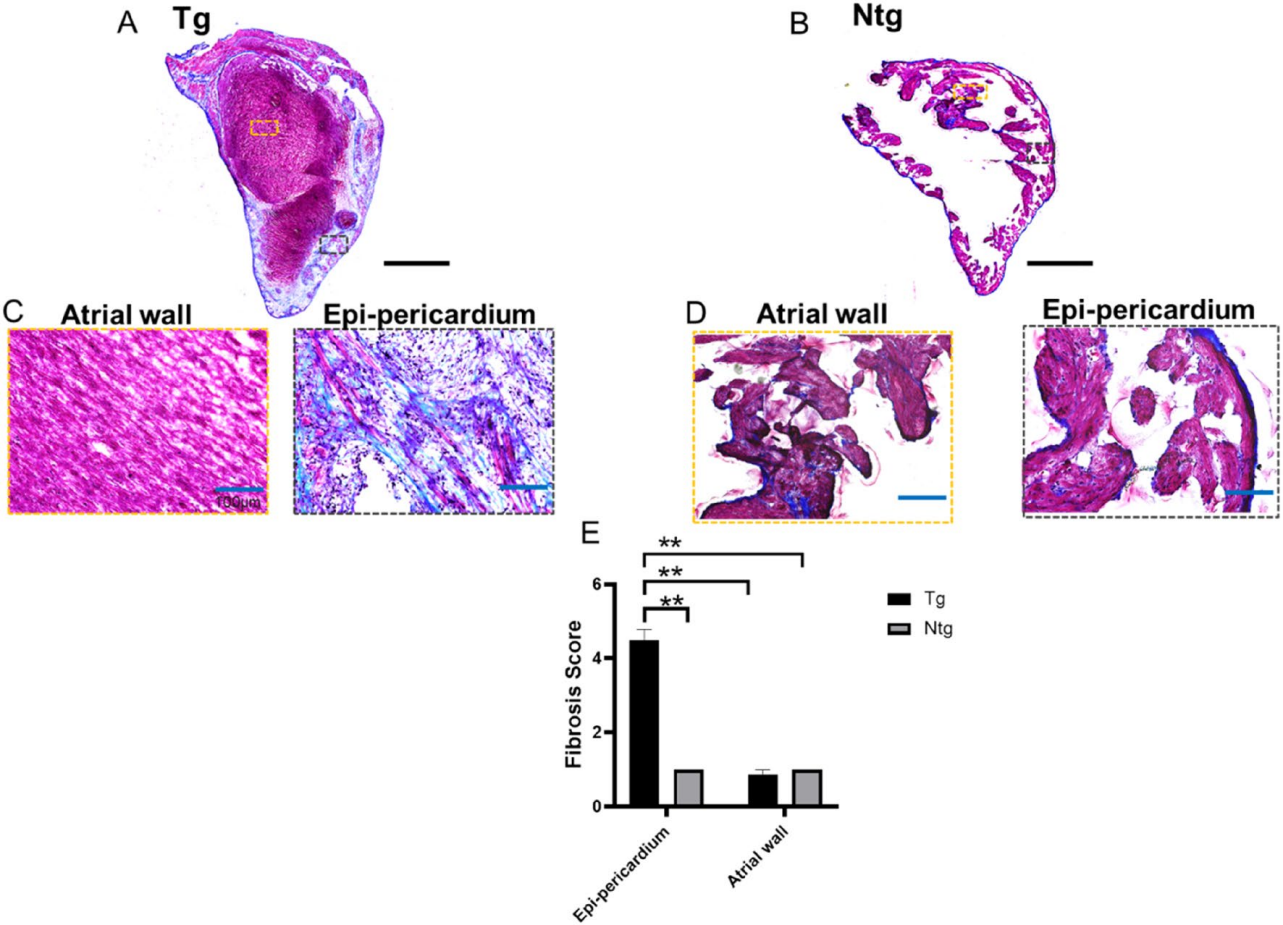
Atrial wall to pericardial fibrosis. Representative images of Masson's trichrome staining of atrial fibrosis in transgenic (Tg) fibrotic (**A**) and non-transgenic (Ntg) non-fibrotic (**B**) animals at 11 months of age. ×10 magnification images of atrial wall and epi-pericardial images of Tg (**C**) and (**D**) Ntg. Ishak fibrosis score of atrial wall to pericardium in Tg and Ntg (**E**). Mean ± SEM. **p < 0.0001 vs. Ntg by 2-way ANOVA followed by post Tukey, Tg; n = 4, Ntg; n = 2; Scale bars for (**A**) and (**B**) = 2000 µm; Scale bars for (**C**) and (**D**) = 100 µm.

**Figure 2:**
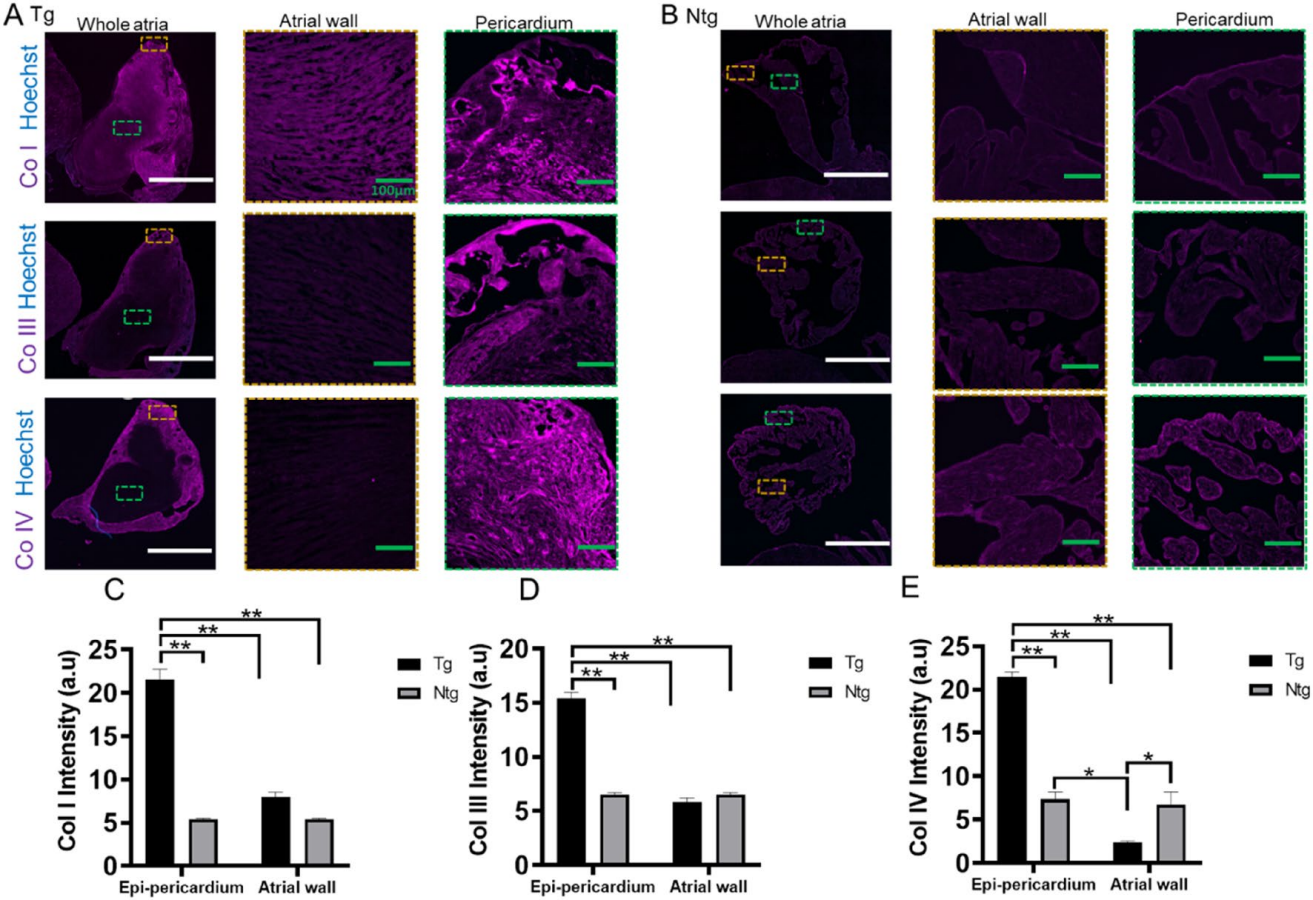
Collagen types in Tg and Ntg mice. Representative atrial wall to epi-pericardial images of immunohistochemical analysis of various collagen types in fibrotic Tg (**A**) and Ntg (**B**) at 11 months of age. Mean fluorescence intensities of collagen I (**C**), collagen III (**D**) and collagen IV (**E**). Mean ± SEM, **p < 0.0001 vs. Ntg. 2-way ANOVA followed by post Tukey, Tg; n = 3, Ntg; n = 2 for collagen I; Tg; n = 4, Ntg; n = 2 for collagen III; Tg; n = 3, Ntg; n = 2 for collagen IV. Scale bars for (**A**) and (**B**) left 1000 µm; Scale bars for (**A**) and (**B**) right = 100 µm.

In both right and left atrial tissue sections from the mice, the epi-pericardial layer was an ‘enlarged’ layer partly irreversibly separated from adjoining atrial myo-endocardium in the transgenic mice (Tg) compared to the wildtype health control (Ntg) (Fig. 1A, B). As shown, to characterize fibrotic remodelling and collagen expression from atrial wall to the epi-pericardium, we did Masson’s trichrome and immunohistochemical stainings. Figure 1A, B reports the atrial tissue section of Tg and Ntg mice on Masson’s trichrome staining, with the epi-pericardial areas of Tg mice highly fibrotic with lesions and loss of normal parallel tissue structural alignment (Fig. 1C right ) compare with that of the Ntg (Fig. 1D right). In contrast to the epi-pericardial areas of Tg mice, the atrial walls of both the Tg and Ntg mice, and epi-pericardial areas of Ntg had no significant fibrotic remodelling (Fig. 1E). We then reveal the expression of respective collagen types by immunohistochemical staining to understand the distribution and abundance of various collagen types from the atrial wall to the epi-pericardium (Fig. 2). Epi-pericardial collagen I, III and IV increased profoundly in Tg compared with both epi-pericardial and myocardial collagen I, III, IV respectively, in Ntg mice (Fig. 2A, C–E). In fact, as per normal atrium, the pericardial area in Ntg tightly encased the atrial wall, without any signs of separation between the layer and the myo-endocardium. Indeed, there was no remodelling between the pericardium and the myocardium in Ntg. Furthermore while Tg atrial wall collagen I had a trend to increase compared with that of Ntg, Tg atrial wall collagen IV was significantly reduced compared with that of Ntg (Fig. 2C, E). There was no observational difference in the epi-pericardial and myocardial collagen in Ntg mice (Fig. 2C–E). In sharp contrast, epi-pericardial collagen I, III and IV were significantly higher compared with atrial wall collagen in Tg (Fig. 2C–E).

### Atrial epi-pericardial-dominant fibrotic mice displayed biatrial thrombi

**Figure 3:**
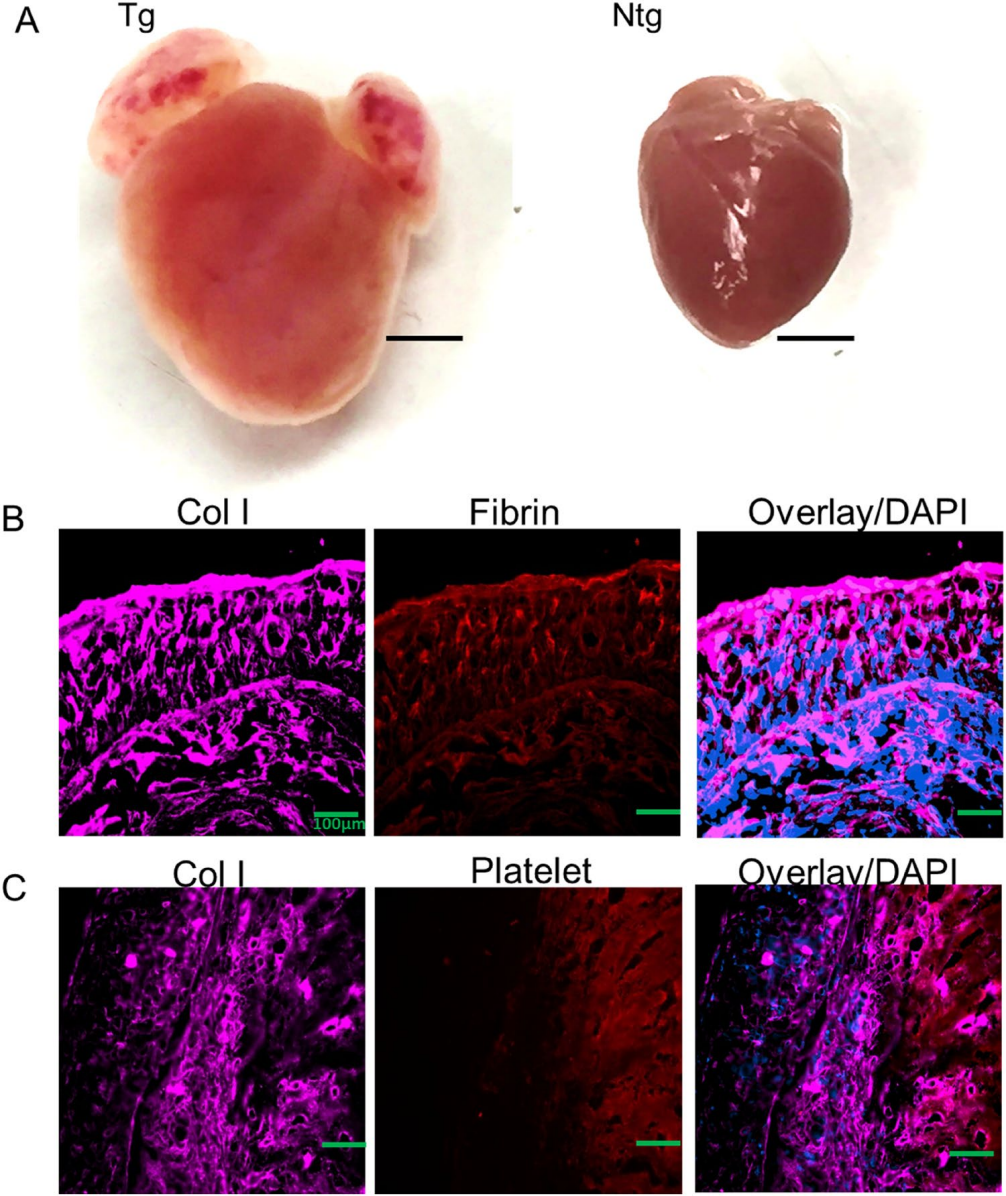
Biatrial thrombi and associated mechanism in Tg. Enlarged four chambers and biatrial thrombi in Tg but not in Ntg (**A**). Type I collagen and fibrin colocalisation (**B**). Type I collagen in association with platelet. Scale bars for (**A**) = 2000 µm; Scale bars for (**B**) and (**C**) = 100 µm.

A cornerstone of therapy for patients with heart failure is β-adrenergic receptor blockers^13^, of which arrhythmia is the major cause of death in patients with heart failure. To gain better insight into transgene dose-dependent response of β_2_-AR expression in heart failure in mouse heart, Liggette et al., generated a series of cardiac-specific β_2_-AR transgenic mice with different doses^14^. The authors found a spectrum of phenotypes from the expression as a critical consequence of level of the doses. While 25-week-old β_2_-60 (a dose of 60-fold overexpression of β_2_-AR) mouse had normal heart size and no phenotypic abnormalities, 25-week-old β_2_-350 (another dose of 350-fold overexpression of β_2_-AR) mouse had 4-chamber enlargement with severe left ventricular dilatation, atrial mural thrombi and aggressive or delayed cardiomyopathy^14^. Histological examination of the hearts revealed severe fibrotic replacement of left ventricular myocardium, left atrium enlargement with extensive laminar thrombus and left ventricle enlargement and hypertrophy^14^. Consistently, 11 months old β_2_-AR mice grossly had severe 4-chamber enlargement, and biatrial thrombi compared with its littermate control (Fig. 3A). While these recapitulate the cardiomyopathy features reported in Liggett et al., atrial epi-pericardial fibrotic lesion and biatrial thrombi has not been previously reported. Furthermore, given that the epi-pericardial layer apparently appears distended from the atrial wall (Fig. 1A compared with Fig. 1B), which has not been reported in normal atria, we report irreversible dilation of the atria in β_2_-AR mice at 11 months of age (Fig. 3). These myopathy features are important and open up a new research outlook for AF pathogenesis.

To explain the biatrial thrombi we thought that the epi-pericardial dominant fibrotic remodelling can effect myo-endothelial lining and expose sub-endothelial collagen to provoke thrombosis, independent of atrial rate. Build-up of type I fibrillar collagen increases fibrin turnover and accumulation, and glycoprotein IIb activation and platelet aggregation as hallmarks of thrombus generation. Therefore in an attempt to characterise thrombotic propagation as a result of collagen accumulation, we determined the relative contribution and local distribution of thrombus contents: fibrin and platelets, in relation to type I collagen deposition by immunohistochemical co-staining in the transgenic mice. Images obtained from the staining showed deposition of fibrin onto type I collagen (Fig. 3B), with areas of platelet aggregation enveloped by type I collagen (Fig. 3C). Overlay images of fibrin and type I collagen demonstrate clear colocalisation, while the pattern for platelets was an association; clearly demonstrating thrombogenic potential. Though the result was not shown in real-time imaging of thrombogenic propagation, in line with previous studies^15,16^, it hints that atrial platelet–fibrin-dependent thrombus generation requires collagen exposure, as a determinative triggering process. Whether this is a high atrial rate clot or tissue-driven architectural process has not been established. Further studies will use novel in vivo methods to demonstrate this in atrial disease.

**Figure 4:**
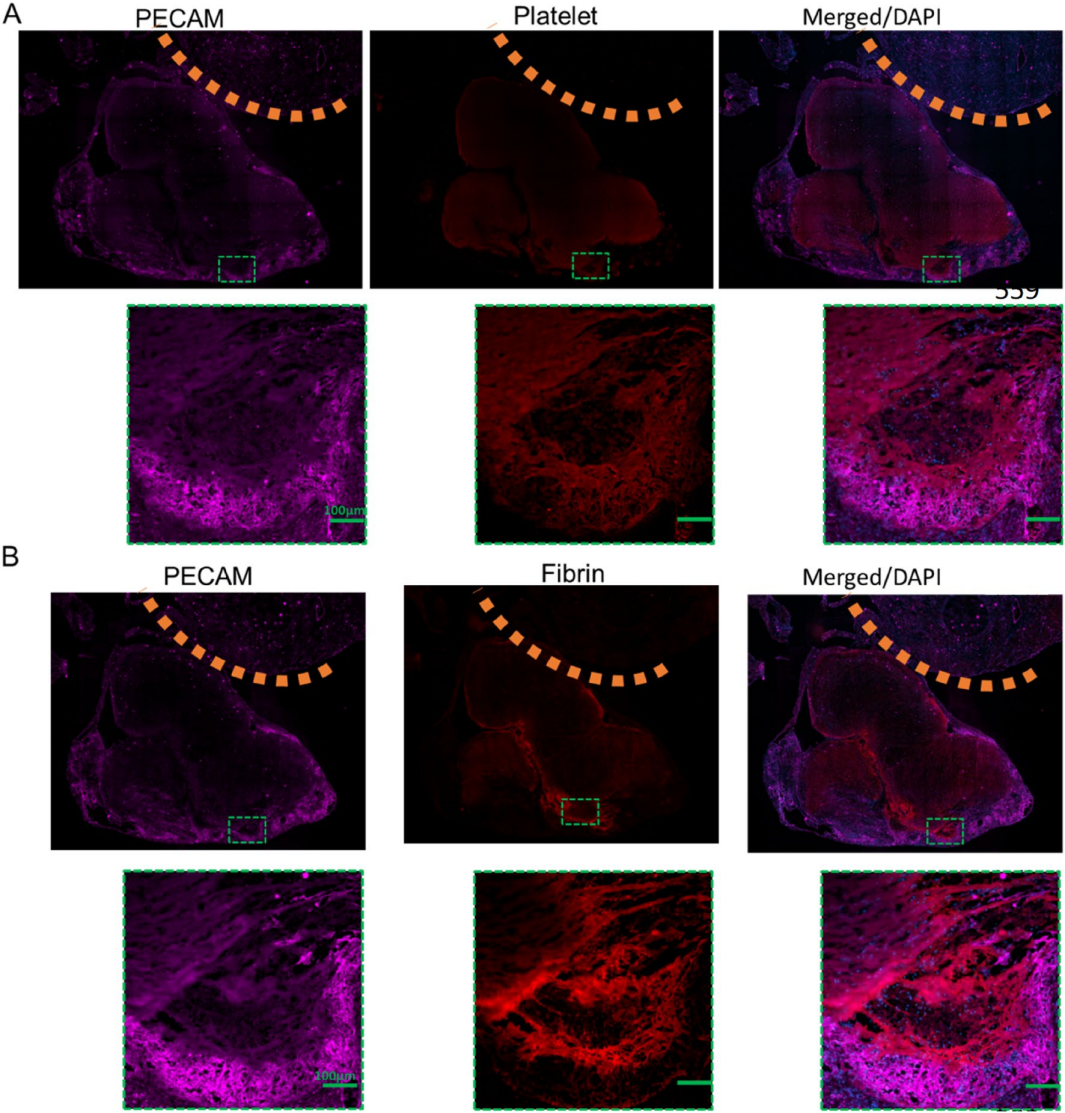
Endothelial Cell Clustering and Embolic events in Tg. Overlay of PECAM-1 and platelet (**A**) and overlay of PECAM-1 and fibrin (**B**), associated with atrial emboli. Scale bars = 100 µm.

To demonstrate the emboli on the basis of tissue-driven architectural process, the elucidation of some of the signals that drive endothelial cell clustering and remodelling leading to the thrombogenic potential of platelet–fibrin-dependent thrombus generation upon collagen exposure will enable better understanding of how the heart reorganises their cellular and extracellular components in response to embolic events. The endocardial endothelial cell surface is exposed to constant blood flow, and as such can provide a sensory stimulus to clot formation during a focal atrial epi-pericardial remodelling. Based on the vital obligatory role of platelet endothelial cell adhesion molecule-1 (PECAM-1), as an endothelial cell surface marker, in deciphering such signals in endothelial cells^17^, we evaluated the clustered immunohistochemical expression of endocardial PECAM-1, as an indication of endothelial cell damage or rather pro-thrombotic state for clot development in the β_2_-AR mice. The immunohistochemical staining revealing the expression of PECAM-1 expectedly showed dramatic areas of PECAM-1 clusters (Fig. 4), indicating degenerative inner lining of the heart and emboli, as demonstrated in extravasated emboli in mice brain pathology using confocal imaging^18^. The clusters were overlaid by platelet (Fig. 4A) and fibrin (Fig. 4B). Importantly, the overlay was only present in the atria, but not in the ventricles (top, indicated by dotted lines (Fig. 4A, B).

Aside from the theory of sub-endothelial collagen exposure to provoke thrombosis independent of atrial rate, inflammation is a known feature of cardiomyopathies. From the initial characterization of Elster, Braunwald and Wood in the 1950s, HF is inundated with pro-inflammatory systemic factors, such as C-reactive protein^19^. It is now appreciated that these factors also include angiotensin II, aldosterone, noradrenaline, adrenaline and atrial natriuretic peptide^20^, due to high neurohormonal stimulation. Therefore, in the cardiac-specific β2-AR mice, it is possible that systemic levels of these factors triggered a systemic response to the failing heart, including the atria, which can consequently trigger clot formation. Inflammation and left atrial endothelial dysfunction have been shown in a rat model after a distinct generation of early right and left insular stroke^21^. Furthermore, for better classification and in recognition of AF detection after stroke or transient ischemic attack, the diagnosis of atrial fibrillation post-stroke has been recently used^22^. This said, in the present study, it is important to note that this second theory was not investigated, suggesting the presence of an already existing ventricular myocardial neurohormonal response in the atrial pericardial pathology.

### Ex vivo peptide enhancement and traditional Masson’s trichrome staining of atrial pericardial collagen

From the above epi-pericardial remodelling investigations, focal atrial pericardial fibrotic calcified lesion was gleaned (Fig. 2). Therefore, we next employed collagen I and MMP-cleaved collagen IV tracers (Cyclic peptide and T-peptide) that target these collagens with better avidity^12,23,24^. The aim of which is to detect the focal atrial pericardial fibrotic lesion, and quantify interstitial fibrosis, as arrhythmogenic substrate during cardiomyopathy by fluorescence-based approach of the tracers.

**Figure 5:**
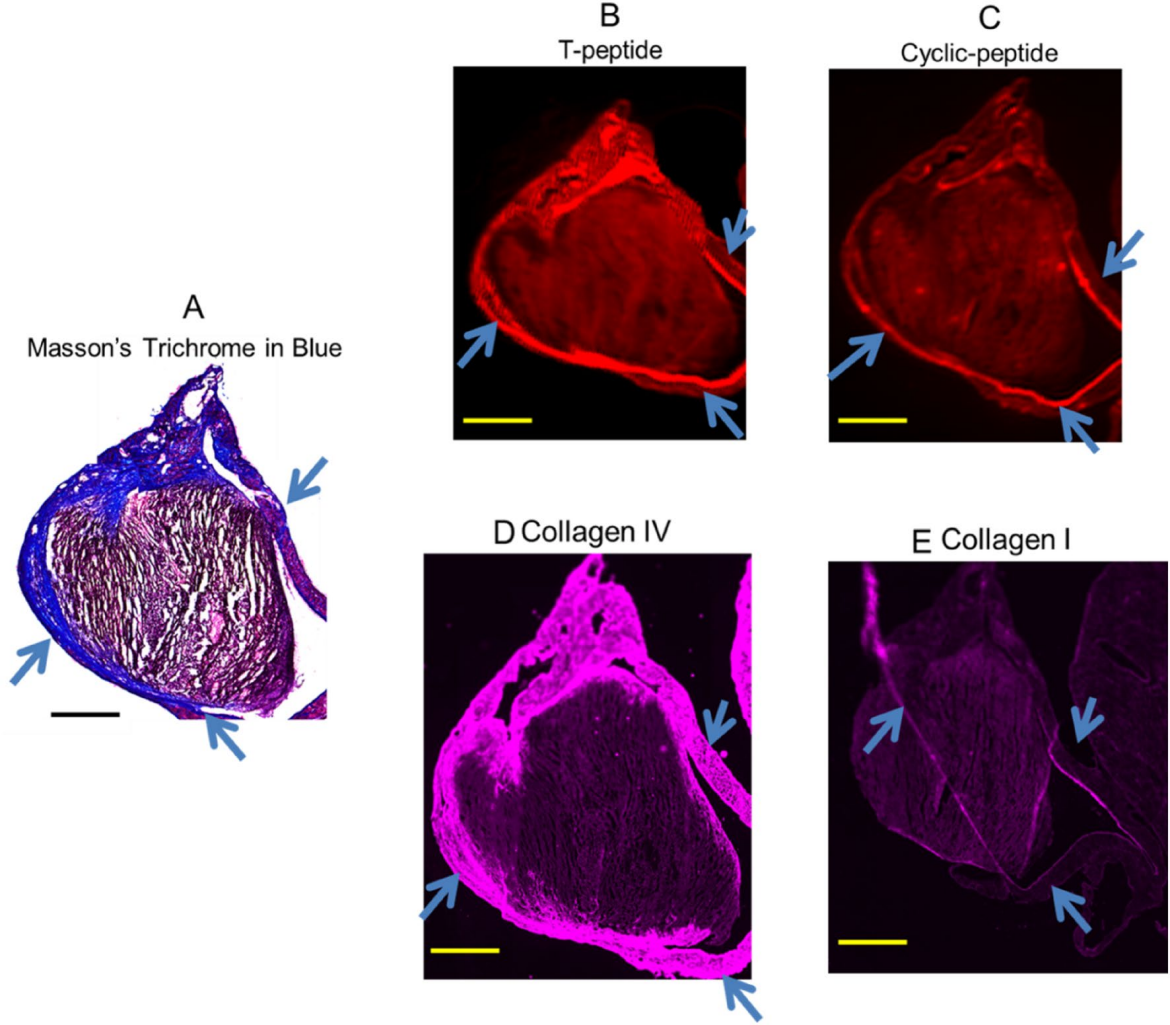
Traditional Mason’s trichrome staining (**A**) correlated qualitatively with T-peptide probe (**B**), cyclic peptide probe (**C**), type IV collagen immunofluorescence (**D**) and type collagen I immunofluorescence (**E**). T-peptide targets collagen IV while cyclic peptide probe targets collagen I. It is important to note that every normal atrium has intact pericardium tightly fastened to the wall of the working atrium. Tg; n = 2, Ntg; n = 2; Scale bar for (**A**) = 2000 µm; Scale bars for (**B**) and (**C**) = 1.5 mm. Blue arrow indicates disassociating pericardium.

**Figure 6:**
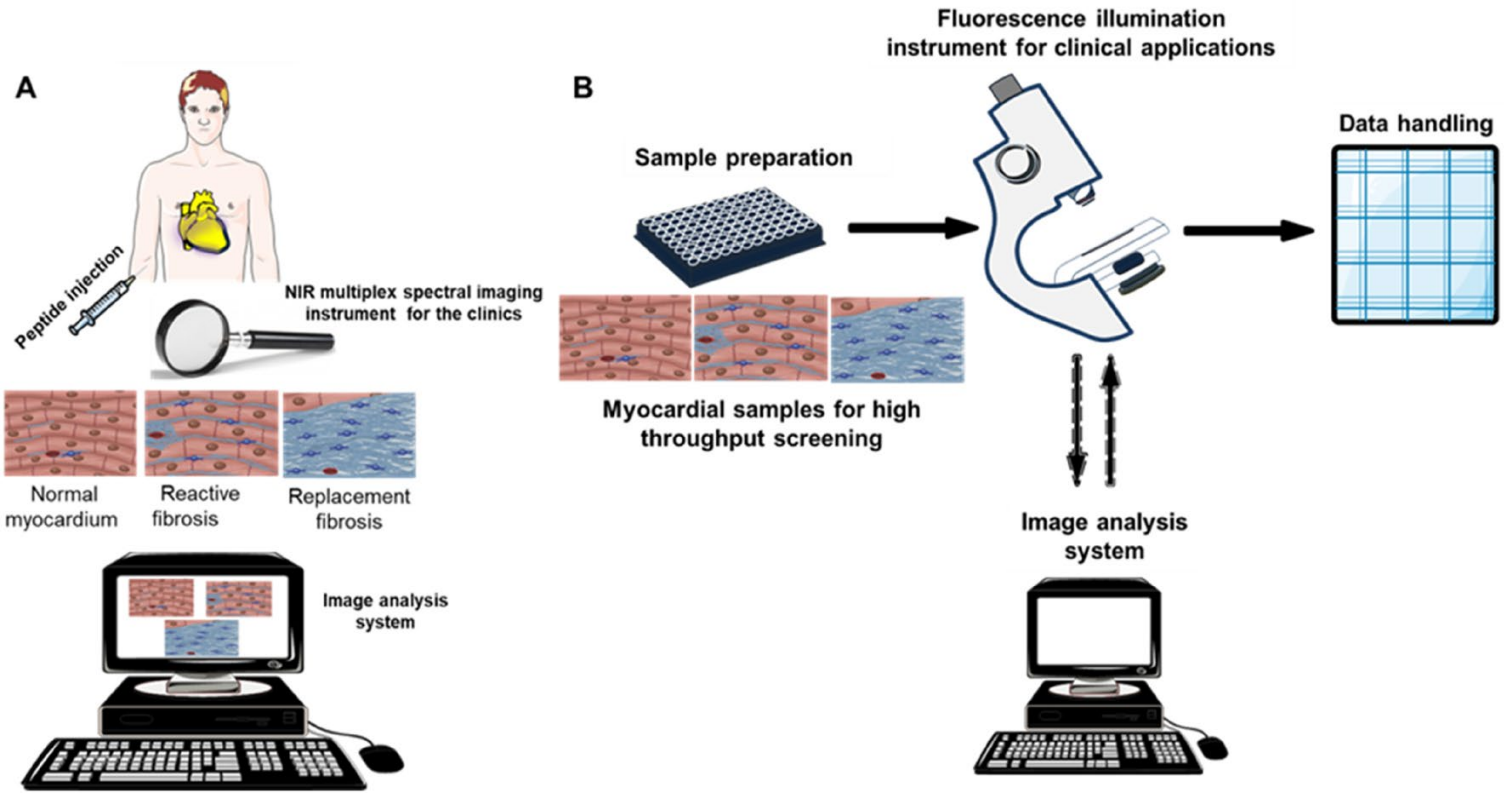
Schematics illustration of near-infrared (NIR) fluorescence imaging of atrial fibrosis for in vivo and ex vivo clinical applications. (**A**) An in vivo method would require the injection of a targeted collagen peptide tracer into disease-affected and healthy individuals for NIR multiplex spectral imaging detection and analysis by a dedicated/customised analysis system. (**B**) An ex vivo approach would involve preparation of excised myocardial specimens from cardiac patients for investigation by high throughput screening, coupled with quantitative fluorescence microscopy.

Fluorescence reflectance imaging as well as non-nuclear imaging proves to be important clinical approaches in cardiology arena to non-invasively detect atrial disease^6,25,26^. Ex vivo sulfo-Cy5.5)-peptide pericardial collagen staining was performed by incubating atrial tissue sections in the optical materials at room temperature. This was done to compare areas of collagen enhancement on the sections to that of the traditional Masson’s trichrome staining (Fig. 5A). In 4 h incubation of the sections in sulfo-Cy5.5 labelled T-peptide, enhanced pericardial collagen, blue arrows, dense red line (Fig. 3B), corresponded with the areas of the Masson’s trichrome staining (Fig. 5A), blue arrows. Similarly, incubation of Cy5.5 labelled cyclic peptide enhanced collagen areas of the pericardium, blue arrows, dense red line (Fig. 5C), and yielded a corresponding staining pattern to Fig. 5A, as annotated in blue arrows, but to a less degree, in terms of intensity, compared with that of T-peptide. Because T-peptide and cyclic peptide target type IV and I collagen, respectively, we did immunofluorescence staining of type IV and I collagen to confirm, their targeting by the peptide probes. Consistently, the stained areas on the immunofluorescence (blue arrows and dense purple lines; Fig. 5D, E) corresponded to the areas of probes staining (blue arrows and dense red lines; Fig. 5A, B). Notably, endomyocardial fibrosis by Masson’s trichrome staining better correlates with extracellular volume analysis by CMRI-LGE in cardiac amyloid patients^27^. Indeed, our ex vivo results also correlated with the traditional Masson’s trichrome staining. Together, our findings suggest that these peptide probes can target and identify pericardial collagen. Therefore, the development of in vivo peptide imaging tools is required, as shown by the summary illustration (Fig. 6) for roles in clinical application. These modalities can also include radioactive imaging by positron emission tomography (PET).

### High-throughput preclinical assessment of atrial collagen enhancement by the peptides

**Figure 7:**
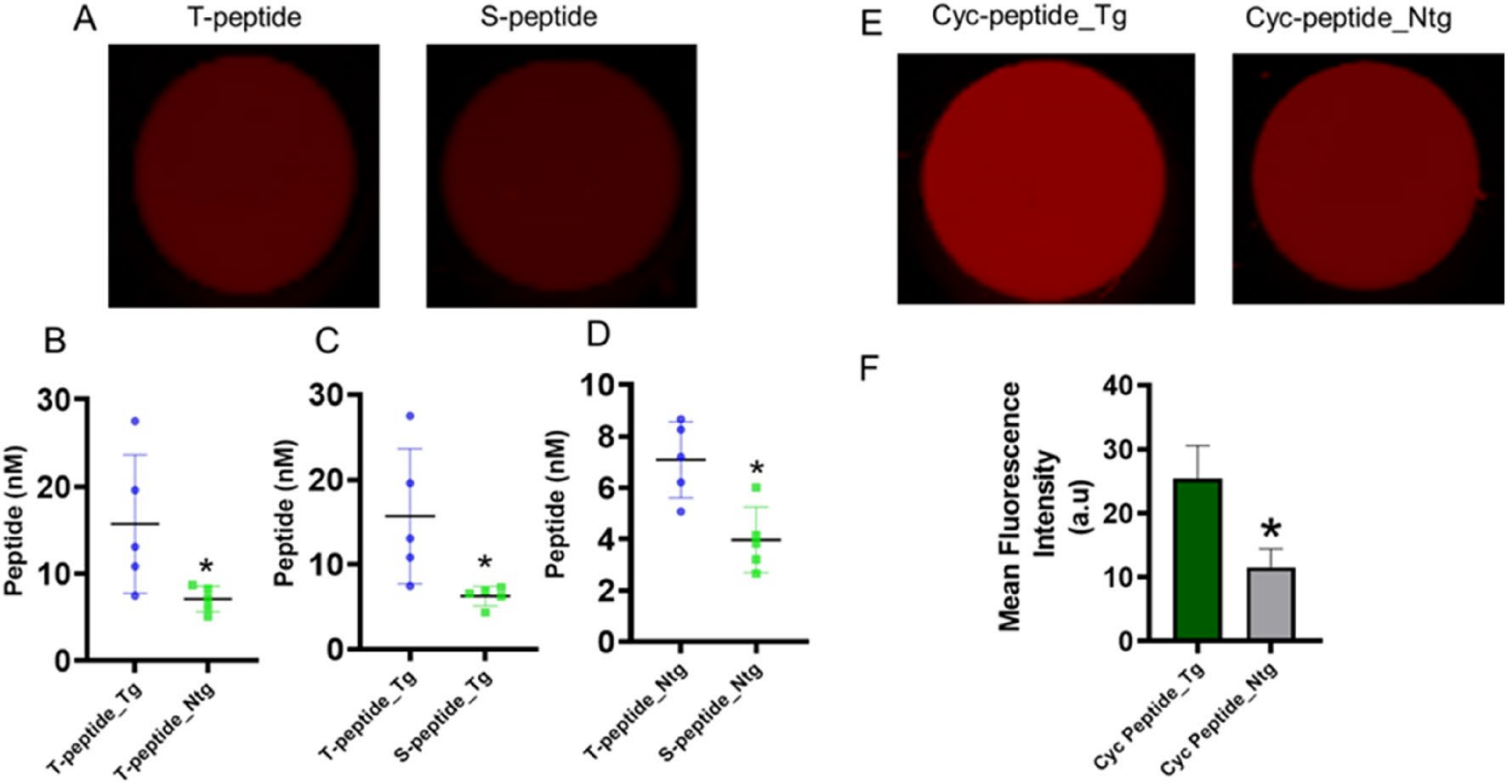
Ex vivo Odyssey imaging of peptide enhancement of collagen in clarified MAL. Representative well-plates of the 2-D Li-Cor scan showing T-peptide enhancement in the MAL (**A**). Measurement of the T-peptide collagen enhancement in the MAL (**B**). Measurement of the T-peptide specificity in the enhancement at the disease level (**C**). Measurement of the T-peptide specificity in the enhancement at the basal level (**D**). Representative well-plates of the 2-D Li-Cor scan showing cyclic peptide enhancement in the MAL (**E**). Measurement of the cyclic peptide collagen enhancement in the MAL (**F**). Mean ± SEM, *p < 0.05 vs. Tg by unpaired *t*-test; n = 5.

To assess the retention of the peptides by collagen I and IV in the pericardium, and atrial wall, and together quantify interstitial collagens, we conducted high-throughput screening and quantification of type I and IV collagen signals in mouse atrial lysate (MAL). 4 h after intravenous injection of the tracers, β_2_-AR mice upper chamber of the heart was obtained, homogenised and clarified. Up clarification by centrifugation, the MAL for T-peptide probe and S-peptide probe (mutant control version of T-peptide) were sampled on a near-infrared NIR 2-D scanner (Li-Cor) for odyssey imaging (Fig. 7A). Apparently, the peptides enhanced odyssey imaging demonstrated greater uptake of the peptides significantly in the Tg MAL compared with Ntg MAL (Fig. 7B).

The fluorescence reflectance in specificity of the uptake of T-peptide at disease (Fig. 7C) and basal (Fig. 7D) levels was also significant. For cyclic peptide, we adopted the T-peptide’s study design and procedure, and compared mean fluorescence uptake of the cyclic peptide, which was high in Tg MAL compared with Ntg MAL (Fig. 7E, F). Taken together, given the traditional collagen staining pattern, which showed that the atrial wall of transgenic animals had little or no collagen IV, unlike collagen I, this result shows that the tracer can be suitable for reporting pericardial collagen in the atrial wall. Our data exemplify the possibility of fluorescence imaging to visualize atrial fibrosis by means of molecular tracers. In particular, the findings hold promising implication for right ventricular imaging (as we have previously shown^12^), which is especially challenging given the thin nature of structures in the right heart. Notably, the right ventricle is larger in volume than its left counterpart, but has smaller mass and thinner wall, which impedes CMRI-LGE imaging.

## Discussion

The pericardium encasing the myocardium is well known for its bio-mechanical and lubrication effects. It contains a fibrous sac and purposes immune cells/functions^28^. Here, we assessed atrial wall to epi-pericardial fibrosis in mice with cardiac-specific, chronic activation of β_2_-AR, to determine changes in various collagen types and their abundance, as a potential marker of atrial myopathy. This was performed with an aim to identify atrial pericardial collagen during pericardial enlargement and fibrosis of the atrial wall, both as components of the arrhythmogenic substrate, using a novel optical-based probe imaging. By revealing the expression of the respective collagen type from the atrial wall to the epi-pericardium, we demonstrated for the first time that mice with cardiac-specific, chronic activation of β_2_-AR can have atrial pericardial pathology, such as extensive pericardial fibrosis, enlargement, disorganised tissue alignment and disassociation of the epi-pericardium and/or even the pericardium alone (Fig. 5) from the atrial myocardium, leading to irreversible dilation. Therefore, chronic activation of β_2_-AR in heart failure is a previously unidentified squealer of pericardial pathology in mice. The mechanism of a focal, atrial-pericardial pathology was not investigated and could be associated with inflammatory response, as has been shown in pericardial adipose tissue of patients with AF^29^.

The mice equally had biatrial thrombi. The presence of thrombi may not be explained by AF and could be related to heart failure, given chronic activation of β_2_-AR signalling^30^. Atrial myopathy is an indicator for AF, which coexists with heart failure and thromboembolism^31^. Chronic activation of β_2_-AR signalling in heart failure can lead to atrial myopathy and AF, but whether this is causal is not completely known. Our findings, together with the increasing evidence connecting AF and pericardial fat, offer a new research area on the pathogenesis of AF**.**

### Pericardial tissue disease as an atrial myopathy leading to AF

Given that for AF to occur, a trigger, a substrate, and the activation of the nervous system should coexist^32,33^, one could speculate that pericardial tissue might contribute to AF by the formation of a substrate, a trigger and the activation of the intrinsic cardiac autonomic system. The abundance of pericardial fibrosis, as demonstrated by this study, the dense sympathetic and parasympathetic innervation of the pericardial space^34^ and the pericardium as a regulator of cardio-bio-mechanical properties, point to: (1) the possible role of cardiac pericardial tissue in atrial fibrosis, (2) the autonomic tone modulation and (3) triggering of arrhythmia, respectively. Furthermore, we think that a complex interplay occurs between several components during the known pathobiology of β_2_-AR activation in the ventricular myocardium^35^, including cardiac inflammatory cytokines, reactive oxidative species and adipokines, which can contribute to the fibrotic remodelling of the atrial myocardium. More so, losing pericardial double-layer harmonic continuity due to subpericardial fibrocalcified lesions can be a key determinant of fibrillatory circuitry.

### New imaging approach for atrial-pericardial, fibrotic, arrhythmogenic substrate detection

A history of postoperative AF heightens suspicion for pericardial pathology and myopathy because of inflammatory state^36^. Animal models of atrial pericardial disease are required to delineate the mechanisms, but they are limited, and the common denominator of essence in cardiovascular disease is fibrosis. Knowledge on the interplay between this and AF will depend on advances in imaging tools to detect and improve the ability to visualise subcellular, cellular, molecular, tissue and/or organ components. Cardiac MRI is a non-invasive tool that uses a magnetic field and radiofrequency to create detailed anatomical pictures and detect abnormalities. It is becoming a tool of choice to detect myocardial fibrosis in numerous cardiovascular conditions^37^, including AF reoccurrence^7^, and compared with localised, invasive procedure provides better accurate diagnosis, because of reduced sampling error. With its exquisite anatomic detail and soft tissue characterisation, MRI is one of the most resourceful modalities to probe the pericardium, which has been used for imaging-guided diagnosis, management and therapy of pericardial diseases^38–40^. While this is well known for the lower chamber of the heart, it is less so for the atria.

However, irrespective of the area or chamber, the demonstration of replacement fibrosis by LGE is an indicator of non-metabolically active (adverse or rather near non-viable tissue) prognosis^41^. LGE cannot probe settings where fibrosis is diffused, reactive and microscopic. In addition, in pericardial settings particularly, disease conditions may usually not be associated with mortality, meaning that autopsy correlation with pre-mortem MRI could be non-existent. For instance, cardiac MRI detects pericardial inflammation^39,40^, but direct confirmation of this with histological data is lacking. New imaging modalities are therefore needed to detect and quantify myocardial fibrosis. Here, we describe potential optical materials to detect atrial-pericardial and interstitial fibrosis in the clinic. Importantly, we previously reported based on histological examination and rapid blood clearance sampling in mice that the peptides were not toxic^12^. However, peptides biotoxicity assessment could further benefit from whole blood biochemical analysis, analysing electrolyte levels and liver function after peptide administration.

This study shows that the novel T-peptide and cyclic peptide tracers labelled with cyanine 5.5 detect atrial pericardial fibrosis using optical imaging. We first showed that it is feasible to detect T-peptide- and cyclic peptide-mediated collagen enhancement of the pericardium after β2-AR chronic signalling. The high accumulation of the tracers at regions of pericardial fibrosis provided excellent target-to-background contrast. Furthermore, Tg MACL had better T-peptide and high cyclic peptide collagen enhancement compared with Ntg MACL for atrial wall fibrosis. We then assessed whether the T-peptide uptake was specific at both basal and disease levels and found uptake specificity in both conditions. Importantly, fibrosis constitutes the etiology of many cardiovascular diseases and subclinical detection of fibrosis is crucial for both diagnosis and intervention. Our probe therefore offers new avenues for pericardial/subclinical detection of fibrosis, diagnosis and management of AF.

## Conclusion

Though our study is in animals, which may not completely correlate to the clinical setting, and the mechanisms of atrial pericardial fibrosis as a denominator of atrial myopathy leading to AF remains to be investigated, we demonstrated the feasibility of optical imaging of pericardial and interstitial fibrosis using the new optical materials. These materials may enable non-invasive visualization and quantification of fibrosis and represent promising candidates for translation in clinical investigation.

## Materials and methods

### Study approval

Ethics approval for animal experimentation was obtained from the Alfred Research Alliance (ARA) Animal Ethics Committee (project E/1941/2019/M). All methods were carried out in accordance with the relevant guidelines and regulations (Australian code for the care and use of animals for scientific purposes). All methods are reported in accordance with ARRIVE guidelines. No studies were conducted on human subjects or human tissues.

### Peptide synthesis and characterisation

Our novel T-peptides was discovered by phage display technology and synthesized by solid phase peptide synthesis techniques^24^. A heptapeptide library was displayed on Enterobacteria phage and intravenously injected into mice bearing Lewis Lung Carcinoma. 10 min after the injection, tumour-bound phages were harvested. This phage pool was then panned in vitro on immobilized MMP-2-degraded collagen IV from the human placenta. Seven peptide sequences were finally extracted, including the sequence TLTYTWS (T-peptide), which binds to MMP-2-degraded collagen IV, but not to native collagen IV^24^. The peptide was conjugated with NIR dye sulfo-Cy5.5. A sulfo-Cy5.5-conjugated mutated binding isomer, termed ‘‘S-peptide’’, was also generated as a control to test the specificity of the T-peptide.

The cyclic peptide was synthesized by peptide synthesis, followed by labelling with sulfo-Cy5.5. The cyclic peptide was previously shown to be effective for collagen I targeting^23,42–44^ and was evaluated for in vitro collagen affinity^23^. For supplemental information on reagents and methods see^12^.

### β_2_-AR Tg mouse model

Alpha myosin heavy chain (α-MHC) promoter directs cardiac-specific expression of transgenes to cardiomyocytes. This promoter was used to target the expression of human β_2_-AR in the mouse heart. In detail, the human β_2_-AR coding sequence was cloned under a murine α-MHC promoter and delivered by pronuclear microinjection into embryos that were then surgically introduced into pseudo-pregnant C57Bl/6 female mice^45^. This transgenesis provides the opportunity to examine (and potentially target) the role of β_2_-AR in cardiac pathogenesis. The model produces classical phenotypes, such as fibrosis and dilated cardiomyopathy (DCM), and has important clinical relevance^14^. Nonetheless, it also has some disadvantages, because dose-dependent transgenesis can lead to dose-related phenotypes^46^. For instance, while enhanced transgenic expression levels of even non-toxic proteins can lead to cardiomyopathy and increased cardiac contractility, modest expression levels may not achieve this effect^45^. However, it is important to note that our transgenic mice express constant levels of the β_2_-AR transgene and therefore do not have variable cardiac phenotypes.

### Odyssey acquisition

To assess the uptake of the peptides in the atria, the peptides were injected intravenously into the mice at 0.5 mg/kg of body weight. 4 h post-injection, animals were humanely euthanised by an overdose of ketamine (300 mg/kg) and xylazine (30 mg/kg), and transcardially perfused with 20 ml of physiological saline. The atria were harvested for analysis. Peptide fluorescence intensity was measured on the Odyssey NIR 2-D scanner (Li-Cor). Analysis of peptide fluorescence intensity was done with Image Studio 5.2 software by defining a region of interest and obtaining mean and fluorescence intensity values.

### High throughput mouse MAL screening for peptide collagen enhancement

FastPrep-96™ high throughput bead beating grinder was used to obtain MAL from the upper chamber of the heart. The atria were obtained, and homogenised based on wet/weight (mg/ml) of the tissues in PBS in 0.5 mL tubes (attached cap, cat no 5076100) containing 200 mg of lysing matrix D (cat no 6983–001) from MP Biomedicals. The tissues underwent two cycles of homogenisation at 1800 CRF, 45 s each, and modified thereafter with 1× RIPA buffer (0.22% Beta glycerophosphate, 10% 4-Nonylphenol, branched, ethoxylated, 0.18% Sodium orthovanadate, 5% Sodium deoxycholate, 0.38% EGTA, 1% Sodium lauryl sulfate, 6.1% Tris, 0.29% EDTA, 8.8% Sodium chloride, 1.12% Sodium pyrophosphate decahydrate), then placed on an orbital rocker for 1 h and kept frozen at −80 °C, which was thawed and clarified by centrifugation at 1600 CRF for 10 min in the cool room. 55ul of supernatant was sampled and scanned in 96-well plates on Li-Cor.

### Peptide ex vivo collagen staining

Specimens were obtained and sectioned at 10 µm. Slides were washed 2×, 7 min each in PBS at room temperature to remove optimal cutting temperature (OCT) material. Sections were then blocked in 10% normal goat serum (NGS) for 2 h. The NGS was aspirated and the sections were then incubated in 0.038 µg/ml and 0.188 µg/ml of T-peptide and cyclic peptide respectively for 4 h at room temperature. Sections were then washed 3×, 5 min each and mounted with ProLong™ Diamond Antifade and scanned on a Li-Cor scanner.

### Immunohistochemistry and immunofluorescence

Fresh (unfixed) isolated hearts with atrial prep were embedded in moulds filled with optimal cutting temperature (OCT) and frozen on dry ice. Tissues were then kept at − 80 °C until sectioning. 10 μm cryosections were obtained and stained by Masson’s trichrome and immunofluorescence. For the latter, sections were first incubated for 1 h at room temperature in a blocking solution (10% normal goat serum in PBS). Anti-collagen type I, collagen type III and collagen type IV primary antibodies [SC-59772, ab7778 and ab6586, respectively, diluted 1:100 (10 µg/ml) in blocking solution] were incubated with the sections overnight at 4 °C. The sections were then washed 3 times for 5 min each in PBS and incubated with Alexa Fluor® 647 rabbit anti-mouse or goat anti-rabbit secondary antibodies for 3 h at room temperature [diluted 1:1000 (2 µg/ml) in blocking solution]. For co-staining of type 1 collagen with platelet or fibrin, a rabbit anti-collagen type I antibody was co-applied overnight at 4 °C with either rat anti-mouse CD41 (BD Biosciences) or Alexa Fluor® 546-conjugated mouse anti-fibrin antibody, clone 59D8 [made by the Monash Antibody Technologies Facility and labelled by Ms Volga Tarlac, Monash University], respectively, all diluted in blocking solution. For collagen 1 with platelets, sections were then washed 3 times for 5 min each in PBS and incubated with both Alexa Fluor® 647 goat anti-rabbit and Alexa Fluor® 568 donkey anti-mouse IgGs (Thermo Fisher) for 3 h at room temperature at 1:1000 dilution (2 µg/ml). For collagen 1 with fibrin, sections were incubated with Alexa Fluor® 647 goat-anti rabbit IgG (note that the anti-fibrin antibody was directly conjugated, as mentioned above). The co-staining of Platelet endothelial cell adhesion molecule-1 (PECAM-1) with fibrin or platelets was performed in a similar manner with a rabbit anti-human PECAM-1 antibody (cross reactive to mouse PECAM-1; produced and kindly provided by Prof Robert Andrews and Prof Elizabeth Gardiner, Australia) that was revealed with Alexa Fluor® 647 goat anti-rabbit antibody. Nuclei were counterstained by addition of Hoechst (5 µg/ml) for 1 h (2 h post-application of the secondary antibody). The sections were finally washed 3 times for 5 min each in PBS and mounted on slides using ProLong™ Diamond Antifade mountant (ThermoFisher). Images were acquired on a widefield fluorescence and confocal microscopes (Nikon Ti-E and Nikon A1r, respectively, controlled by the NIS Elements imaging software), at 20× magnification and identical settings for each collagen type. Quantification of mean signal intensity was calculated using Image J after thresholding.

### Statistical analyses

Analyses were done using GraphPad Prism 8. Results are presented as mean ± standard error. *t*-tests were used to compare two groups for a single measure. Differences between 3 or more groups were analysed using a two-way analysis of variance (ANOVA) followed by Tukey’s post-hoc. A *p*-value under 0.05 was considered significant.
